# Poly(ethylene glycol) Diacrylate Hydrogel with Silver Nanoclusters for Water Pb(II) Ions Filtering

**DOI:** 10.3390/gels9020133

**Published:** 2023-02-04

**Authors:** Luca Burratti, Marco Zannotti, Valentin Maranges, Rita Giovannetti, Leonardo Duranti, Fabio De Matteis, Roberto Francini, Paolo Prosposito

**Affiliations:** 1 Department of Industrial Engineering, University of Rome Tor Vergata, Via del Politecnico 1, 00133 Rome, Italy; 2 Department School of Science and Technology, Chemistry Division, ChIP Research Center, University of Camerino, Via Madonna delle Ceneri, 62032 Camerino, Italy; 3 Department of Chemical Science and Technologies, University of Rome Tor Vergata, Via Della Ricerca Scientifica 1, 00133 Rome, Italy

**Keywords:** silver nanoclusters, fluorescent nanoclusters, poly(ethylene glycol) diacrylate, PEGDA hydrogel, hybrid material, water remediation, heavy metal ions filtering, Pb(II) ions

## Abstract

Poly(ethylene glycol) diacrylate (PEGDA) hydrogels modified with luminescent silver nanoclusters (AgNCs) are synthesized by a photo-crosslinking process. The hybrid material thus obtained is employed to filter Pb(II) polluted water. Under the best conditions, the nanocomposite is able to remove up to 80–90% of lead contaminant, depending on the filter composition. The experimental results indicate that the adsorption process of Pb(II) onto the modified filter can be well modeled using the Freundlich isotherm, thus revealing that the chemisorption is the driving process of Pb(II) adsorption. In addition, the parameter *n* in the Freundlich model suggests that the adsorption process of Pb(II) ions in the modified hydrogel is favored. Based on the obtained remarkable contaminant uptake capacity and the overall low cost, this hybrid system appears to be a promising sorbent material for the removal of Pb(II) ions from aqueous media.

## 1. Introduction

Heavy metals pollution has become one of the most serious environmental issues nowadays. Pb(II) ions are commonly present in many types of industrial effluents and are responsible for environmental contamination. The toxic effect and bioconcentration of lead ions represent an important issue for the health of many ecosystems [1,2]. Lead is a particularly hazardous heavy metal because once it enters the human body, it disperses immediately and causes harmful effects wherever it lands. For instance, Pb(II) can accumulate in many organs, such as the kidneys, adversely affecting the nervous, immune, reproductive, and cardiovascular systems [3,4]. Lead toxicity also impacts vegetation health as it reduces leaf growth by decreasing the level of photosynthetic pigments, alters the chloroplast structure, and decreases the enzymatic activity of CO_2_ assimilation [5,6]. Many industrial processes, such as battery manufacturing, pigments and printing, metal plating and soldering materials, ceramic and glass industries, and iron and steel manufacturing units, are the main sources of lead contamination in wastewater [7,8]. Limiting lead pollution is of paramount importance for human health, as well as environmental and economic considerations.

The most commonly used techniques to eliminate heavy metal ions (HMIs) from water are represented by adsorption, membrane-based filtration and separation, chemical separation, and electric-based separation [9].

In the adsorption process, the adsorbent material, through chemical interaction with the functional groups (such as carboxyl, phenyl, etc.) on its surface, can adsorb HMIs. The materials principally used as adsorbents are carbon-based materials [9,10], which sometimes are very expensive. Mineral adsorbents, such as zeolite, silica, and clay, show a high cation exchange capacity [11], and synthetic materials, such as MOFs (metal-organic frameworks), which show stability in water and toxicity, can be a serious problem for the depuration of polluted water [12].

Other techniques involve the use of membranes for the filtration and the extraction of HMIs, such as Pb(II), using membrane pores larger than the HM ions; in this case, additives bonded to metal ions to enlarge the size can be used, while ultrafiltration and polymer enhanced ultrafiltration are developed [13,14,15]. Chemical methods involve the use of hydroxides and sulfides that allow for HMI precipitation; in this case, the main problem is the great dosage of precipitating agent and the large volume of sludge [16]. Coagulation and flocculation methods are also used, followed by a sedimentation process in order to obtain clean water [17,18]. Finally, electrochemical methods such as electrochemical reduction, electrocoagulation, electroflotation, and electrooxidation can also be used [19,20].

Among all methods, adsorption is the most used and studied due to its ease of operation, low cost, and high adsorption capacity. The adsorption method can be divided into two types depending on the type of interactions between the adsorbent and adsorbate (pollutant): chemisorption and physisorption. Chemisorption is an irreversible process where the driving force is the strong chemical interaction between the adsorbate and adsorbent surface [21]. Conversely, physisorption is a reversible process where weak intermolecular physical forces are involved between the adsorbate and adsorbent, such as π-π and dipole–dipole interactions, van der Waals forces, hydrogen bonding, etc. [22]. Several factors, such as the surface area of the adsorbent, temperature, pH, type of interactions between adsorbent and adsorbate, contact time, adsorbent dosage, etc., significantly influence the adsorption efficiency and affect the removal of pollutants from effluent [23].

Adsorbent materials can be divided into several categories [24]: gels (hydrogels, aero-gels), layered nanomaterials (nanoclays, layered double hydroxides (LDH)), carbon nanomaterials (fullerene, graphene), nanoparticles (metal nanoparticles, magnetic nanoparticles), polymer-based nanomaterials (biopolymers, conjugated polymers), and conventional materials (activated carbon, silica gels), and it is also possible to combine two materials to increase the adsorption performances [25,26,27]. Noble metal nanomaterials such as nanoparticles [28,29] or fluorescent nanoclusters [30,31,32] are already employed in many fields of science and engineering [33,34,35,36,37] thanks to their different properties from those of bulk metal [38,39], but only recently they have been combined with adsorbent matrices to boost the adsorption of water pollutants [40,41,42,43].

To synthesize the hydrogel adsorbent matrix, many materials are available; among these, cellulose is available, which is the most abundant natural polymer on Earth. Cellulose has different useful features, such as biocompatibility, biodegradability, non-toxicity, good mechanical properties, etc. [44,45]. The synthesis of the cellulose-based hydrogel consists of several steps and chemical processes as described in the literature [46,47], including copper(I) as a catalyst [48]. Nonetheless, the employment of poly(ethylene glycol) derivatives such as poly(ethylene glycol) diacrylate (PEGDA) represents a valid alternative to the classic cellulose-based hydrogels. PEGDA is a synthetic biopolymer widely used in different fields of research [49,50,51], and its low cost and ease of hydrogel synthesis by photo-polymerization techniques (one-step process) make it suitable for a variety of practical applications. Moreover, these techniques can be easily scaled up to synthesize 3D scaffold hydrogels with different dimensions and shapes [52,53].

In the present work, we synthesized adsorbent materials based on poly(ethylene glycol) diacrylate with silver nanoclusters capped with poly(methacrylic acid) [AgNCs-PMAA], and we compared their properties with those of the unmodified matrix. For matrix synthesis, a photo-polymerization process was exploited using a UV source to activate the photoinitiator (Irgacure^®^ 184), which reacts with the acrylate moieties of PEGDA and cross-linking the polymeric chains. The modified matrix was obtained by mixing the previously synthesized AgNCs-PMAA with PEGDA and Irgacure^®^ 184 before the UV exposition. The two types of hydrogels (with and without AgNCs) were characterized by UV-Vis absorption, emission spectroscopy, and scanning electron microscopy (SEM).

The main aim of this study was to improve the removal efficiency (RE) towards Pb(II) ions of unmodified PEGDA hydrogel by adding the AgNCs-PMAA. The adsorption capacities for both types of hydrogels have been evaluated and compared. In addition, we performed equilibrium studies using different isotherm models, which show an improvement in the RE for the AgNCs-modified filters.

## 2. Materials and Methods

### 2.1. Chemicals

Silver nitrate (AgNO_3_), poly(methacrylic acid) sodium salt water solution (PMAA, MW = 9500, 30% in wt), poly(ethylene glycol) diacrylate (PEGDA, Mn = 700), ethanol (>99.8%), nitric acid HNO_3_ (70%), and lead nitrate [Pb(NO_3_)_2_, > 99.0%] were purchased from Sigma-Aldrich. The photoinitiator Irgacure^®^ 184 (Irg.184) was purchased from Ciba Specialty Chemicals. All reagents were used without any further purification. All the water solutions were prepared with deionized water with resistivity equal to 18.2 MΩ∙cm (Semplicity^®^ UV, MERCK, Darmstadt, Germany).

### 2.2. Synthesis of Silver Nanoclusters (AgNCs-PMAA)

Silver nanoclusters were synthesized according to the previous papers [54,55], where a fresh water solution of AgNO_3_ was prepared and mixed with a solution of PMAA. The pH was adjusted to reach the value of 4 by adding HNO_3_. A volume equal to 3.5 mL was poured into a Petri dish and exposed to strong UV radiation (300 W, NEWPORT, Oriel Instruments, Irvine, CA, USA.) for 6 min to promote the reduction reaction of silver ions to silver metal [56,57]. To hamper the AgNCs surface oxidation, UV exposition took place under a flux of nitrogen gas. The colloidal solution was then purified by centrifugation for 20 min at 10,000 rpm (Thermo Scientific, Heraeus Megafuge 8, Waltham, MA, USA) and was applied in order to obtain, as far as possible, a monodispersed solution of AgCNs. Only the supernatant solution was collected and subsequently used for the synthesis of PEGDA/AgNCs-PMAA filters. The final solution was kept in the dark at T = 4 °C before use. 

### 2.3. Synthesis of PEGDA/AgNCs-PMAA Filters

Filters in the final form were obtained via a photo-crosslinking process under UV irradiation (λ = 366 nm, 1.2 mW/cm^2^ at 10 cm of distance, MinUVIS, DESAGA company, Germany). In a typical synthesis, the Irg.184 was dissolved in ethanol and PEGDA and stirred for 5 min in the dark. Subsequently, the AgNCs-PMAA solution was added dropwise and mixed with the previous precursor solution for 5 min. A volume of 2 mL of the photoactive solution was poured into a square box of 2.7 × 2.7 × 1.0 cm^3^ until complete photo-reticulation. For a given PEGDA to Irg.184 amount ratio, the photo-crosslinking process was optimized by varying the UV exposure time in the range of 2–6 min. Other filters were prepared as references using deionized water instead of the AgNCs solution. In this case, the filters were fully formed after 2 min of UV exposure time. The details of the investigated compositions and exposure times can be found in Table 1.

Before the filtration tests, all hydrogels were cleaned in ethanol for 24 h to remove unreacted Irgacure and PEGDA, followed by 5 days in H_2_O, changing the deionized water every 24 h. Figure S1 of the Supporting Information shows a digital picture of both types (unmodified and modified) of dried filters.

### 2.4. Filtering Tests

Pb(II) filtering tests were carried out in static conditions: a single filter was immersed in a beaker containing 10 mL of Pb(II) polluted water for 24 h and at T = 25 °C. To select the best composition of filter, the quantities of PEGDA (14%, 19%, and 24% in wt.) and AgNCs (from 0 to 255 mg) were varied. These hydrogels were kept in contact with a solution of 1500 ppm of Pb(II) ions, and subsequently, the adsorption percentage was evaluated to select the best composition of sorbent material. The RE percentage was calculated by applying the following formula:

(1) $$RE\;\left(\%\right)=\left(\frac{C_{i}-C_{e}}{C_{i}}\right)*100,$$

where *C_i_* and *C_e_* represent the initial and equilibrium concentrations of metal ion (mg/L), respectively, measured by inductively coupled plasma–mass spectrometry (ICP-MS).

The performance of the filters with the best composition was further investigated by varying the Pb(II) concentration from 1500 ppm to 50 ppm. The same investigation was replicated for the filters without AgNCs for comparison. The number of metal ions adsorbed per unit mass of the sorbent material (mg/g) was evaluated with the following equation:

(2) $$q_{e}=\left(\frac{C_{i}-C_{e}}{m}\right)*V,$$

where *q_e_* (mg/g) is the adsorption capacity at equilibrium; *C_i_* and *C_e_* have the same meaning as in Equation (1), while *V* is the volume of adsorbate solution in liters, and *m* is the mass of the filter in grams. The error analysis is reported in the Supporting Information.

### 2.5. Instrumentation

All filters were dried in an oven at 40 °C overnight before being characterized. Optical absorption in the range of 300–700 nm was measured with a spectrophotometer (Perkin-Elmer, Lambda 750), and the photoluminescence (PL) spectra were obtained with a custom-made apparatus equipped with a LED (Light-Emitting Diode) source peaked at 430 nm with a 17 nm bandwidth (Thorlabs, M430L4). The excitation light was focalized onto the sample surface, and the PL was collected and analyzed by a grating monochromator (ARC, SpectraPro-300i). A long pass filter at 500 nm (Melles Griot, 03LWP003) at the entrance slit prevented the excitation wavelength from entering the monochromator. A photomultiplier tube (Hamamatsu Photonic Corp., R2949) was employed for detection. The output signal was analyzed by a lock-in amplifier (Stanford Research System, model SR830 DSP) in the spectral range of 500–800 nm. A computer running a LabView program controlled the whole setup. A full description of the apparatus can be found in the reference [58].

Morphological analyses were performed using a Zeiss Leo SUPRA 35 field emission scanning electron microscope (FE-SEM), while elemental analysis was performed by energy dispersive X-ray spectroscopy (EDX, INCAx-sight, Model: 7426, Oxford Instruments, Abington, UK). Hydrogels were treated in an oven at 120 °C for 24 h. Once completely dried, samples were cut into 1 mm-thick slices and gold-sputtered prior to the FE-SEM and EDX analyses.

The concentrations of Pb in the filtered water were measured with ICP-MS analysis. All the samples were diluted with an appropriate amount of aqueous HNO_3_ (Supra Pure) 1% *v*/*v*. The measurement was carried out by Agilent Technologies 7500cx Series ICP-MS. The analysis was performed under the following conditions: power 1550 W; carrier gas 1.11 L/min; sample depth 7.5 mm; nebulizer pump 0.1 rps; spray chamber temperature 2 °C. The concentration of Pb was calculated as the sum of ^208^Pb, ^206^Pb, and ^207^Pb.

## 3. Results and Discussion

### 3.1. Optical Characterization

**Figure 1:**
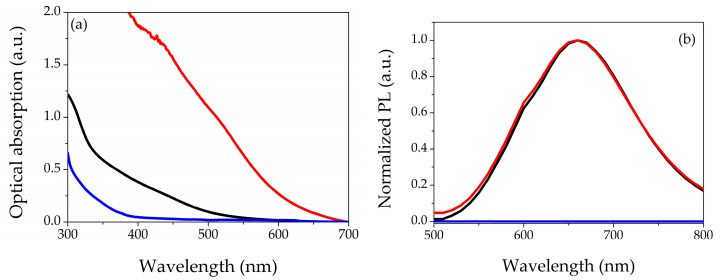
Optical characterizations: (**a**) UV-Vis optical absorption of AgNCs-PMAA colloidal solution (black curve), dried hydrogels with (red line) and without (blue curve) AgNCs-PMAA; (**b**) normalized photoemission spectra excited at λ = 430 nm of AgNCs-PMAA colloidal solution (black curve), dried hydrogels with (red line) and without (blue curve) AgNCs-PMAA.

Figure 1 shows the optical characterizations: Figure 1a represents the UV-Vis absorption spectra of AgNCs-PMAA (black curve), while the red and blue curves refer to the hydrogels with and without AgNCs, respectively. The colloidal solution presents an orange color after synthesis, but its spectrum does not show peculiar features in the analyzed range. The hydrogel with only PEGDA is colorless in the visible range; indeed, there are no absorption bands in this range. The spectrum of the hydrogel with AgNCs-PMAA shows an increasing optical absorption as the wavelength decreases. Figure S1 shows the difference in the color of both filters. In Figure 1b, we report the normalized photoluminescence emission spectra of the three samples in the range of 500–800 nm by exciting at λ = 430 nm. The colloidal solution exhibits an emission band centered at approximately 660 nm with an FWHM of roughly 150 nm (black curve in the graph). The same emission belongs to the NCs-based hydrogel (red curve in the figure), underlining that the insertion of the silver nanomaterial was successful and without modification of the AgNCs’ optical properties. Finally, the hydrogel (blue curve in the graph) shows a null PL. The spectra shown in Figure 1 refer to the hydrogels samples 1–0 and 1-C (see Table 1), and the overall behavior is the same for all the investigated compositions.

### 3.2. Optimization of PEGDA/AgNCs-PMAA Composition

In Table 1, we list the explored compositions and the time needed to polymerize the entire volume poured in the square box. 

**Figure 2:**
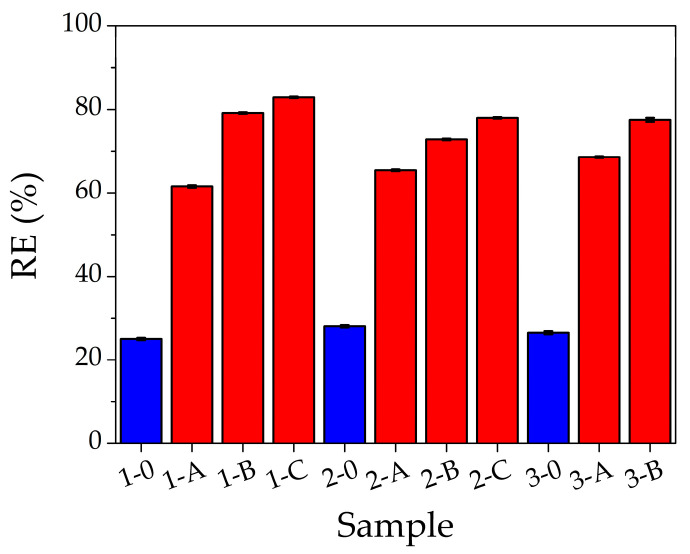
Removal efficiency as a function of filter composition after the filtration of a 1500 ppm of Pb(II) solution. Red bars represent the filters with AgNCs-PMAA, while blue bars correspond to those without AgNCs.

Figure 2 shows the removal efficiency for 1500 ppm of Pb(II) solution as a function of filter composition as listed in Table 1; the red bars represent the filters with AgNCs-PMAA and the blue ones those without nanoclusters. For all PEGDA compositions, the presence of AgNCs inside the filter increases the removal percentage of Pb(II) and the efficiency increases by lowering the PEGDA content. Indeed, the best removal efficiency (83%) corresponds to the filter 1-C. Within the same percentage of PEGDA, by raising the AgNCs content, the Pb(II) adsorption increases. In hydrogels without AgNCs, RE is low and almost constant (on average 26%) for all compositions. The hydrogel with composition 1-C was then selected for the adsorption capacity study.

### 3.3. Morphological and Elemental Characterizations

**Figure 3:**
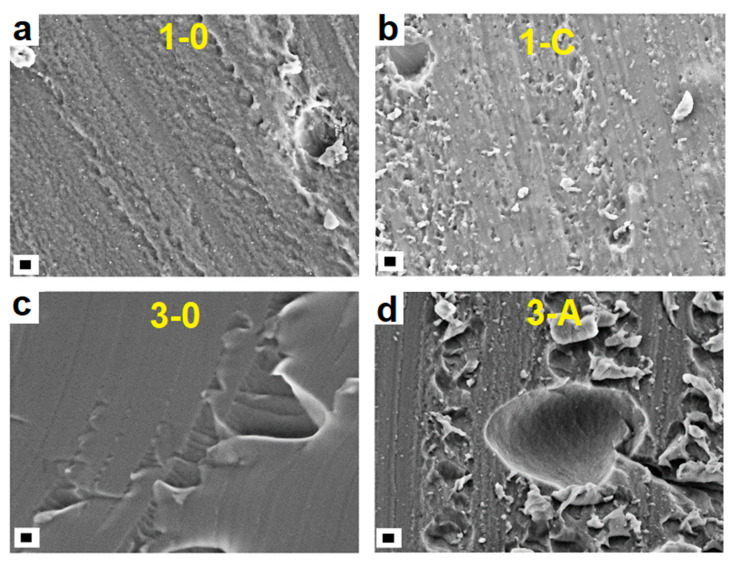
SEM images of (**a**) sample 1–0 with 14%wt of PEGDA and without AgNCs; (**b**) sample 1-C with 14%wt of PEGDA and 255 mg of AgNCs-PMAA; (**c**) sample 3–0 with 24%wt of PEGDA and without AgNCs; (**d**) sample 3-A with 24%wt of PEGDA and 180 mg of AgNCs-PMAA (scale bar 1 µm).

The morphology and the elemental composition of samples with and without AgNCs-PMAA were investigated. The SEM images of modified and unmodified hydrogels are similar and independent of PEGDA percentage, as shown in Figure 3. SEM images at higher magnifications are reported in Figures S2 and S3 of the Supporting Information file. The first two panels of Figure 3 refer to the samples with 14% wt. of PEGDA without silver nanoclusters (1–0, a) and with 255 mg of AgNCs-PMAA (1-C, b). Here, the structures of both hydrogels are similar. The last two panels of Figure 3 display the case of 24% wt. containing PEGDA unmodified (3–0, c) and modified with 180 mg of AgNCs (3-A, d) filters. A similar morphology is present in both cases.

According to SEM analyses, the morphology of the hydrogel does not affect the volumetric adsorption of lead ions, while the presence of AgNCs and especially of the carboxylic groups of the capping agents seems to play a crucial role.

**Figure 4:**
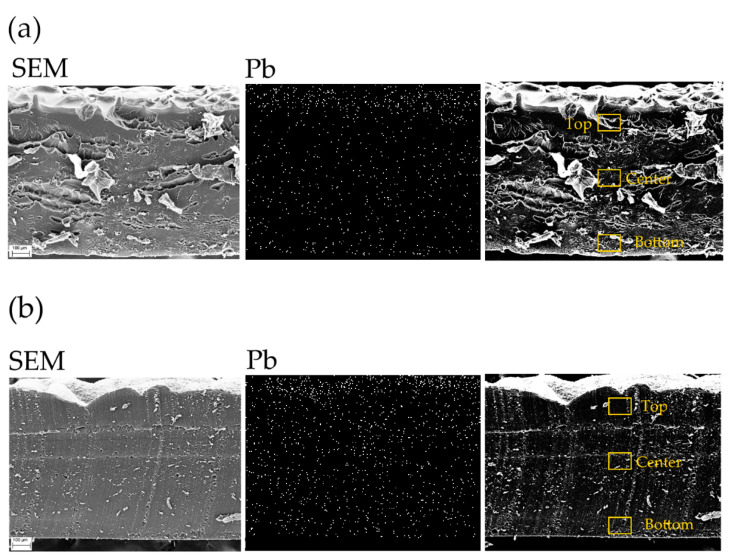
EDX analyses results of 14% PEGDA samples without (1–0, (**a**)) and with (1-C, (**b**)) after the filtration of 1500 ppm Pb(II). For each sample, the SEM image of the recorded area and the Pb distribution map are reported. The yellow boxes in the third panel underline the areas where the localized EDX spectra were acquired. The obtained elemental distributions of localized analyses are reported in Table S1.

EDX analyses were performed on the cross sections of the samples after the Pb(II) filtration process to investigate the element distributions in the hydrogel volume: both on the surface and in bulk. The micrographs of samples 1–0 and 1-C (without and with AgNCs), together with Pb distributions, are reported in Figure 4, while the weight percentages of the detected elements are shown in Table 2. In both hydrogels (without and with AgNCs), the main elements are C and O, as expected, due to the presence of PEGDA (C, O, and Ag EDX maps are reported in Figure S4 of the Supporting Information file). In the case of the unmodified filter, the Pb distribution underlines that the adsorption occurred mainly on the surface of the hydrogel, as shown in the Pb panel of Figure 4a, with a total Pb uptake of 0.97%. In the case of AgNCs-based hydrogel, Figure 4b, the Pb content (9.37%) is approximately one order of magnitude higher compared to that of the filter without AgNCs, as reported in Table 2. The amount of detected silver in the AgNCs-based hydrogel is approximately 1% on average, and Ag distribution appears homogeneous throughout the hydrogel, as shown in Figure S4 of the Supporting Information.

In the AgNCs-hydrogel, the Pb signal shows a high intensity not only on the surface but also inside the bulk of the filter. Thus, indicating that the presence of AgNCs-PMAA plays a key role in the enhancement of the Pb(II) uptake.

By focusing the analysis on three different areas of each sample (top, center, and bottom), the elemental composition was recorded in localized spots of 10 µm width. In the unmodified sample, the local amount of Pb is higher on the top surface (1.77%) than in the rest of the sample (only 0.57% for the center and bottom, see Table S1 of the Supporting Information file for details). This can be explained considering that the top surface was in direct contact with most of the volume of Pb(II) solution during the filtration test, while the bottom part was in contact with the glass of the beaker. On the other hand, in the modified sample, the Pb local distribution is higher at the center (14.50%), as compared to the top (8.43%) and bottom (5.18%). The same trend was observed for silver distribution in the filter (Table S1), showing that, indeed, silver nanoclusters are responsible for the higher Pb(II) uptake.

### 3.4. Adsorption Capacity and Equilibrium Studies

**Figure 5:**
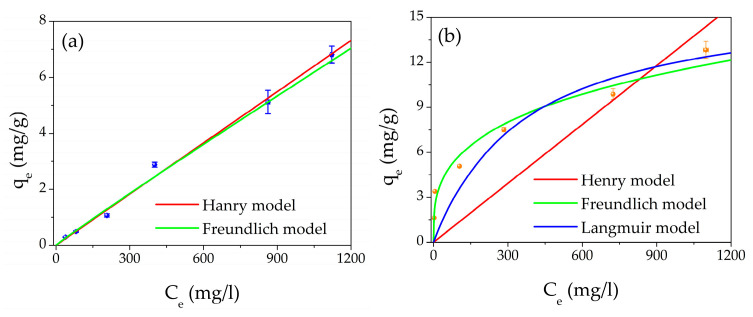
Plot of adsorption capacity (q_e_) as a function of equilibrium concentration C_e_ for: (**a**) AgNCs-free filter, blue points and (**b**) AgNCs modified filter, orange points; the isotherm best fit are also shown in the graphs: red line Henry model, green line Freundlich model, and blue line Langmuir model.

The adsorption capacity study was carried out using different aqueous concentrations of Pb(II) from 50 to 1500 ppm. Figure 5a shows the adsorption capacity as a function of the Pb(II) equilibrium concentration of the unmodified filter. In addition, the best fits of the experimental points are reported: the red line is the Henry isotherm; meanwhile, the green line represents the Freundlich model. Figure 5b shows the adsorption capacity as a function of the Pb(II) equilibrium concentration of the AgNCs-hydrogel filter. In this case, the best fits of the experimental points are reported: the red line is the Henry isotherm, the green line represents the Freundlich isotherm, and the blue curve refers to the Langmuir isotherm. 

The parameters obtained by the best fits are listed in Table 3 for both filter types.

The Henry isotherm is defined as follows [59]:

(3) $$q_{e}=K_{HE}*C_{e},$$

where *K_HE_* represents Henry’s partition coefficient calculated by the slope of the experimental fit. This constant is related to the interaction that occurs among adsorbate and adsorbent, or in other words, how the adsorbate is partitioned between the solution and the filter. The Henry model describes a weak interaction between adsorbate and adsorbent of the electrostatic or van der Waals kind or hydrophobic interactions [59].

The Freundlich isotherm is defined as follows [59]:

(4) $$q_{e}=K_{F}*C_{e}^{\frac{1}{n}},$$

where *K_F_* is Freundlich’s coefficient and is related to the adsorption capacity, while *n* is related to the strength constant of the isotherm model. The Freundlich isotherm model describes a reversible and non-ideal adsorption process and defines the heterogeneity of the surface as well as the exponential distribution of the energies of the active site. Unlike the Langmuir isotherm model, the Freundlich one does not predict the saturation of the adsorbent material; hence, infinite surface coverage is mathematically predicted, indicating that multilayer adsorption on the surface is possible. The Freundlich model describes adsorption of a single layer when chemisorption is the fundamental adsorption mechanism; otherwise, it describes multilayer adsorption when physisorption is the main mechanism [59].

The values of *1/n* $\frac{1}{n}$ depend on temperature and indicate adsorption conditions such as adsorption intensity or surface heterogeneity. The adsorption process is favored if $\frac{1}{n}$ is between 0 and 1, disadvantaged if it is greater than one, and irreversible if it equals one [60].

The Langmuir isotherm is defined as follows [59]:

(5) $$q_{e}=\frac{q_{m}*K_{L}*C_{e}}{1+K_{L}*C_{e}},$$

where *q_m_* (mg/g) is the maximum adsorption capacity, and *K_L_* (l/mg) is the Langmuir isotherm constant. The main point of the Langmuir isotherm assumes that the thickness of the adsorbed layer is one molecule (monolayer adsorption) in which the process of adsorption occurs at identical and equivalent definite localized sites, and the active sites are identical in terms of activation energy.

For the unmodified filter, by considering the R^2^ factors of both models, the best fit is represented by the Henry isotherm (R^2^ = 0.996) compared to the Freundlich one (R^2^ = 0.991). By considering the chemical structure of PEGDA (see Figure S5.a of the Supporting Information), we can suppose that the electrostatic interaction takes place on the oxygen atoms present in the PEGDA molecule.

Regarding the filter with AgNCs-PMAA, from Figure 5b and Table 3, it appears that the best model is the Freundlich isotherm (R^2^ = 0.942). From the experimental points, it is evident that the Henry model (R^2^ = 0.494) does not describe what happens during the interaction between the AgNCs-based filter and Pb(II) ions. Moreover, the Langmuir model (R^2^ = 0.899) does not interpolate the experimental points well, and the precision (defined as the relative error) in the estimation of *K_L_* and *q_m_* is lower than that of *K_F_* and *n*.

Therefore, the best model that describes the adsorption process in the case of AgNCs-based filters is the Freundlich one. In fact, the process is favored (0 < $\frac{1}{n}$ < 1), and the presence of the nanomaterial enhances the filtering effect since the carboxyl groups of PMAA (see Figure S5b of the Supporting Information), which are very close to each other, act as a chelating agent for the Pb ions. The interaction mechanism between Pb(II) and AgNCs-PMAA has already been investigated in previous work [54], where it was reported that the carboxylic groups exactly chelated the ions. In the literature, the chelating action is considered chemisorption [61,62]; thus, we can conclude that the main adsorption mechanism is chemisorption, which is assumed in the Freundlich isotherm. Finally, the values of *K_F_* and *n* obtained from the fit are compared with those of the literature, and they are in good agreement with the values previously found [63,64,65,66].

## 4. Conclusions

In this work, the insertion of AgNCs-PMAA into a PEGDA matrix by a photo-polymerization process was proven. Once inserted in the hydrogel, the AgNCs-PMAA showed the same optical properties as the original colloidal solution, demonstrating that the incorporation took place successfully. The optimization process of the composition revealed that the filters with 14% in weight of PEGDA and 255 mg of AgNCs-PMAA have the best performance by removing 90% of the Pb(II) ions present in the water solution. This value represents an efficiency 60% larger compared to that of the filters without active nanomaterial [RE ≈ 25%, for 1500 ppm of Pb(II) solution]. The study of the adsorption capacity revealed that the Pb(II) adsorption mechanisms are different in the case of unmodified and modified filters with AgNCs. The performed best fits for PEGDA hydrogels suggest an electrostatic interaction between Pb(II) ions and oxygen atoms of the polymeric chains (Henry model). Meanwhile, in the case of PEGDA/AgNCs-PMAA filters, the absorption model follows the Freundlich isotherm, demonstrating that the presence of the nanomaterial enhances the filter effect through the carboxyl groups (from PMAA), giving rise to a strong chelating action towards the Pb(II) ions (chemisorption). As a final remark, the employment of a light-curable polymer such as PEGDA opens new possibilities regarding the synthesis of three-dimensional structured materials to increase the surface interaction between water pollutants and filters.

## Figures and Tables

**Table 1:** Composition of the investigated samples and the UV exposure time.

Sample	PEGDA (% in wt.)	AgNCs (in mg)	UV Exposure Time (min)
1–0	14	0	2
1-A	14	180	6
1-B	14	225	6
1-C	14	255	6
2–0	19	0	2
2-A	19	180	4
2-B	19	225	4
2-C	19	240	4
3–0	24	0	2
3-A	24	180	2
3-B	24	225	2

**Table 2:** Elemental composition of the AgNCs-free and AgNCs hydrogels after the filtration of a 1500 ppm of Pb(II) solution.

Sample	Element	% in Weight
1–0	C	56.00 ± 1.63
	O	42.73 ± 1.71
	Au *	0.30 ± 0.06
	Pb	0.97 ± 0.40
	Tot.	100.00
1-C	C	57.34 ± 3.00
	O	29.92 ± 1.29
	Au *	1.51 ± 0.64
	Na ^†^	0.81 ± 0.26
	Ag	1.04 ± 0.54
	Pb	9.37 ± 2.73
	Tot.	100.00

* The presence of Au is due to the metallization of the samples. ^†^ The Na element is the counter ion present in the PMAA solution; it results from the synthesis of AgNCs.

**Table 3:** Isotherm parameters determined by fitting the experimental points.

Sample	Isotherm Model	Parameters	Sample	Isotherm Model	Parameters
1–0	Henry	*K_HE_* = 6.1 ± 0.4 l/kg	1-C	Henry	*K_HE_* = 13 ± 9 l/kg
		R^2^ = 0.996			R^2^ = 0.494
	Freundlich	*K_F_* = 0.008 ± 0.003 mg/g		Freundlich	*K_F_* = 1.6 ± 0.2 mg/g
		*n* = 1.04 ± 0.06			*n* = 3.4 ± 0.5
		R^2^ = 0.991			R^2^ = 0.942
	Langmuir	Does not converge		Langmuir	K_L_ = 0.05 ± 0.02 l/mg
					q_m_ = 16 ± 3 mg/g
					R^2^ = 0.899

## Data Availability

Not applicable.

## Supplementary Materials

The following Supporting Information can be downloaded at: https://www.mdpi.com/article/10.3390/gels9020133/s1, Figure S1. Picture of dried filters: unmodified PEGDA hydrogel (left); modified with AgNCs-PMAA hydrogel (right). Figure S2. SEM images at different magnifications of samples with 14%wt of PEGDA and without NCs (a)–(d) (from left to right: 10kX scale bare 1 µm, 50kX scale bar 1 µm, 100kX scale bar 100 nm, 500kX scale bar 100 nm); and with 255 mg of AgNCs-PMAA (e)–(h) (from left to right: 10kX scale bare 1 µm, 50kX scale bar 1 µm, 100kX scale bar 100 nm, 500kX scale bar 100 nm). Figure S3. SEM images at different magnifications of samples with 24%wt of PEGDA and without AgNCs (a)–(d) (from left to right: 10kX scale bare 1 µm, 50kX scale bar 1 µm, 100kX scale bar 100 nm, 500kX scale bar 100 nm); and with 180 mg of AgNCs-PMAA (e)–(h) (from left to right: 10kX scale bare 1 µm, 50kX scale bar 1 µm, 100kX scale bar 100 nm, 500kX scale bar 100 nm). Figure S4. Map distributions of chemical elements of hydrogel samples after the filtration of 1500 ppm of Pb(II): (a) unmodified and (b) modified filters. Table S1. Elemental composition of filters as a function of different areas of interest. Figure S5. Chemical structure of PEGDA molecule (a) and PMAA molecule (b).
