# Supplementary material for: Poly(ethylene glycol) Diacrylate Hydrogel with Silver Nanoclusters for Water Pb(II) Ions Filtering
Source: Gels. 2023 Feb 4;9(2):133. doi: 10.3390/gels9020133 (PMC9957228; doi:10.3390/gels9020133)
Supplement: Supplementary file 1 [file gels-09-00133-s001.zip › gels-2190346-supplementary.pdf]

gels MDPI

Supplementary Materials

Poly(Ethylene Glycol) Diacrylate Hydrogel with Silver Nanoclusters for Water Pb(II) Ions Filtering

Luca Burratti [1,*], Marco Zannotti [2,*], Valentin Maranges [1], Rita Giovannetti [2], Leonardo Duranti [3], Fabio De Matteis [1], Roberto Francini [1] and Paolo Prosposito [1]

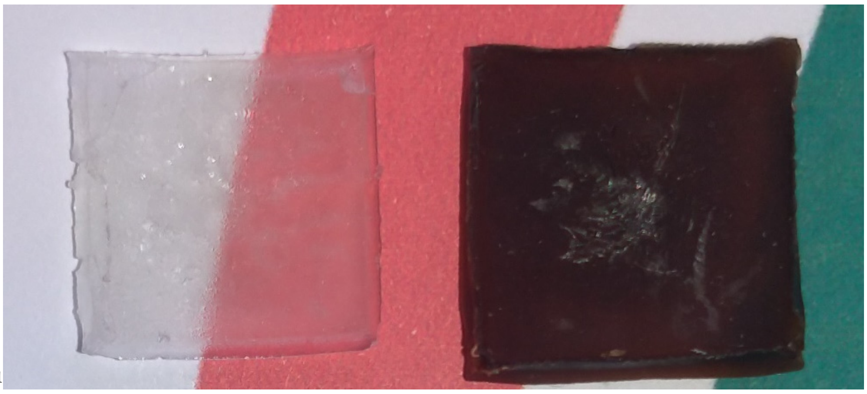

Figure S1. Picture of dried filters: unmodified PEGDA hydrogel (left); modified with AgNCs-PMAA hydrogel (right).

Citation: Burratti, L.; Zannotti, M.; Maranges, V.; Giovannetti, R.; Duranti, L.; De Matteis, F.; Francini, R.; Prosposito, P. Poly(Ethylene Glycol) Diacrylate Hydrogel with Silver Nanoclusters for Water Pb(II) Ions Filtering. *Gels* **2023**, *9*, x. https://doi.org/10.3390/xxxxx

Academic Editor(s): Avinash J. Patil

Received: 13 January 2023
Revised: 25 January 2023
Accepted: 1 February 2023
Published: date

Copyright: © 2023 by the authors. Submitted for possible open access publication under the terms and conditions of the Creative Commons Attribution (CC BY) license (https://creativecommons.org/licenses/by/4.0/).

Error analysis

The error in Removal Efficency (RE) in percentage was calculated according with the general propagation of error, by appling the following formula:

$$\delta_{RE} = \sqrt{\left[\frac{\partial_{RE}}{\partial_{C_i}}\delta_{C_i}\right]^2 + \left[\frac{\partial_{RE}}{\partial_{Ce}}\delta_{C_e}\right]^2} * 100 \qquad (S1)$$

Where δ_{C_i} and δ_{C_e} rapresent the absolute errors of initial and equilibrium concentrations, respectively.

About the error in adsorption capacity (q_e) [i.e. $\delta(q_e)$] was found by applying the general propagation error as follow:

$$\delta q_e = \sqrt{\left[\frac{\partial_{qe}}{\partial_{C_i}}\delta_{C_i}\right]^2 + \left[\frac{\partial_{qe}}{\partial_{Ce}}\delta_{C_e}\right]^2 + \left[\frac{\partial_{qe}}{\partial_V}\delta_V\right]^2 + \left[\frac{\partial_{qe}}{\partial_m}\delta_m\right]^2} \qquad (S2)$$

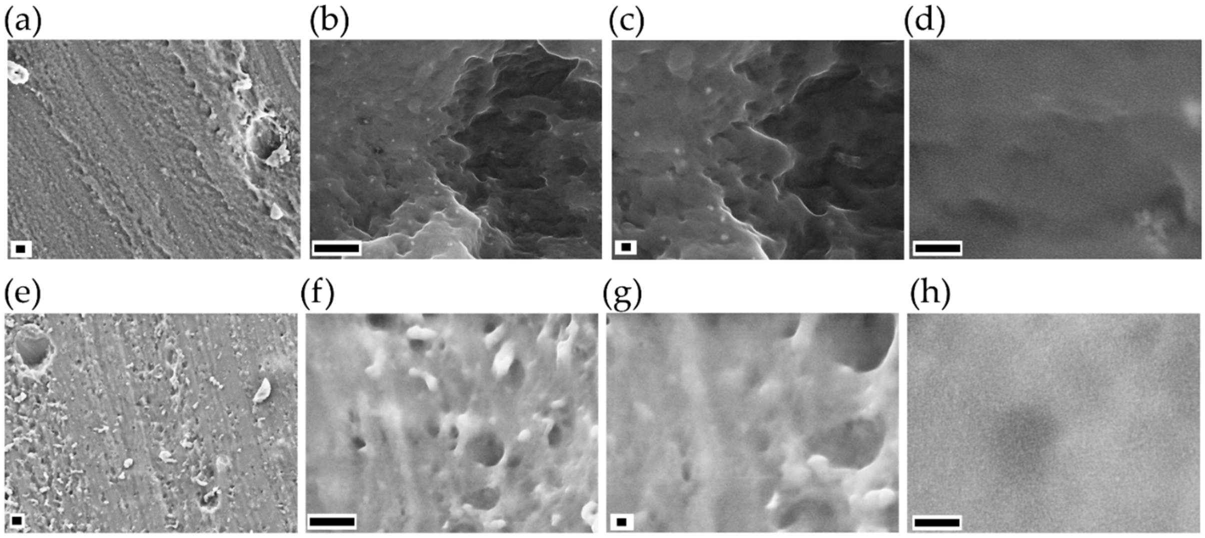

Figure S2. SEM images at different magnification of samples with 14%wt of PEGDA and without NCs (a)-(d) (from left to right: 10kX scale bare 1 µm, 50kX scale bar 1 µm, 100kX scale bar 100 nm, 500kX scale bar 100 nm); and with 255 mg of AgNCs-PMAA (e)-(h) (from left to right: 10kX scale bare 1 µm, 50kX scale bar 1 µm, 100kX scale bar 100 nm, 500kX scale bar 100 nm).

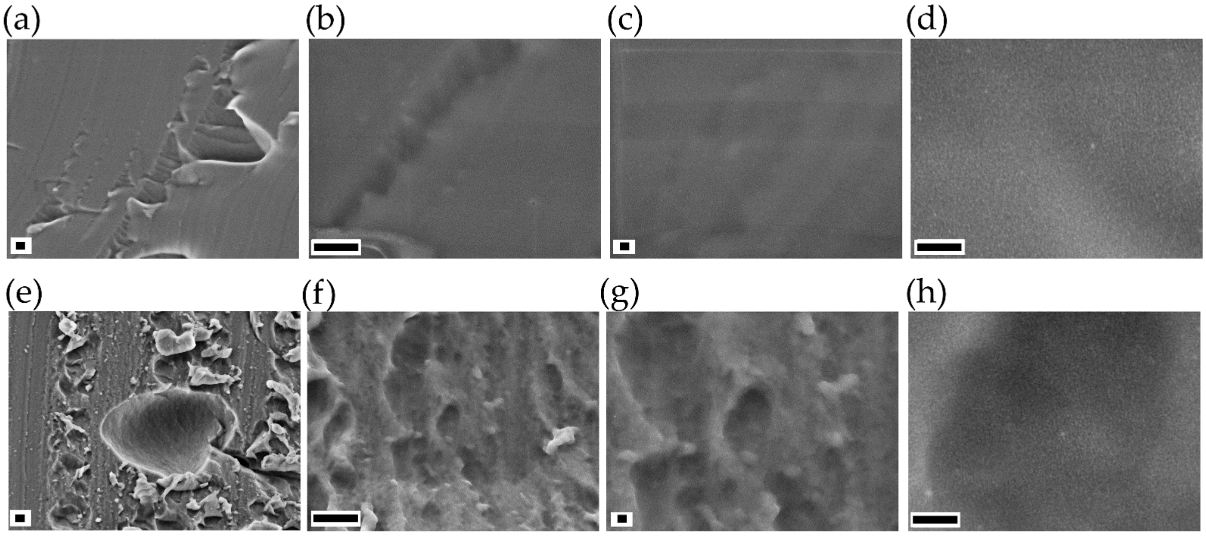

Figure S3. SEM images at different magnification of samples with 24%wt of PEGDA and without AgNCs (a)-(d) (from left to right: 10kX scale bare 1 µm, 50kX scale bar 1 µm, 100kX scale bar 100 nm, 500kX scale bar 100 nm); and with 180 mg of AgNCs-PMAA (e)-(h) (from left to right: 10kX scale bare 1 µm, 50kX scale bar 1 µm, 100kX scale bar 100 nm, 500kX scale bar 100 nm).

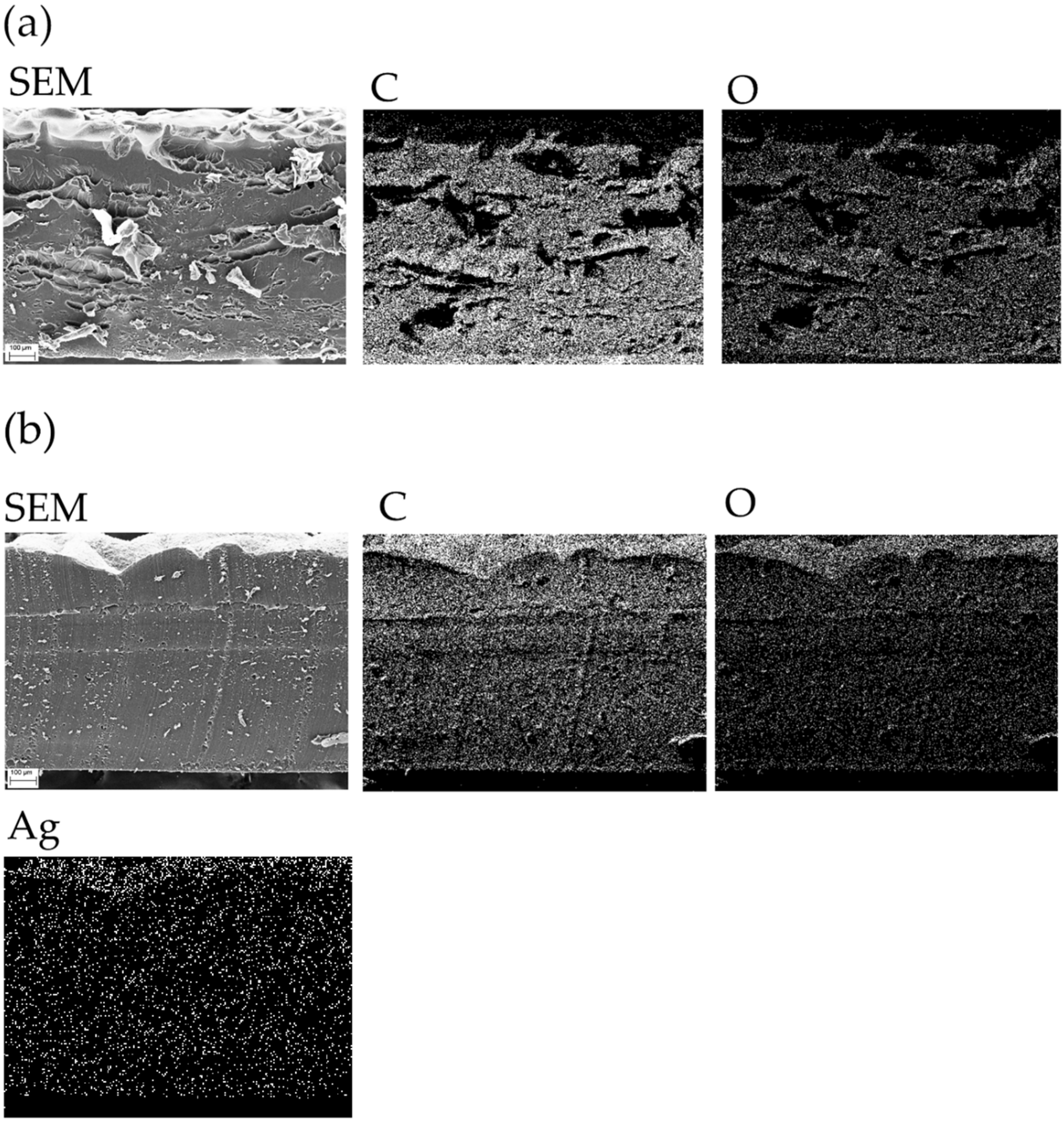

Figure S4. Map distributions of chemical elements of hydrogel samples after the filtration of 1500 ppm of Pb(II): (a) unmodified and (b) modified filters.

Table S1. Elemental composition of filters as a function of the different areas of interest. Samples were sputtered with gold prior to the analyses.

Sample	Area	Element	% in weight	Sample	Area	Element	%in weight
1-0	Top	C	56.45	1-C	Top	C	59.92
		O	41.39			O	29.35
		Au	0.39			Au	1.38
		Pb	1.77			Na	0.53
		Tot.	100.00			Ag	0.40
	Center	C	52.98			Pb	8.43
		O	46.13			Tot.	100.00
		Au	0.32		Center	C	51.36
		Pb	0.57			O	28.03

	Tot.	100.00		Au	2.68
	C	58.56		Na	1.32
	O	40.67		Ag	2.11
Bottom	Au	0.20		Pb	14.50
	Pb	0.57		Tot.	100.00
	Tot.	100.00		C	60.75
				O	32.39
				Au	0.48
			Bottom	Na	0.58
				Ag	0.61
				Pb	5.18
				Tot.	100.00

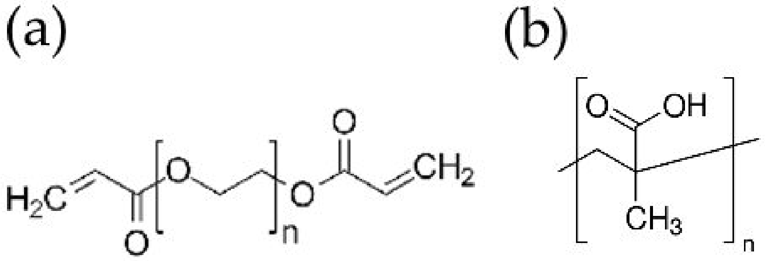

Figure S5. Chemical structure of PEGDA molecule (a) and PMAA molecule (b).
